# A Fatal Case of Vancomycin Associated Drug Reaction with Eosinophilia and Systemic Symptoms Syndrome in a Septuagenarian

**DOI:** 10.7759/cureus.5015

**Published:** 2019-06-27

**Authors:** Mounika Gangireddy, Manbeer S Sarao, Isha Shrimanker, Vinod K Nookala

**Affiliations:** 1 Internal Medicine, University of Pittsburgh Medical Center - UPMC - Pinnacle, Harrisburg, USA; 2 Internal Medicine, Griffin Hospital, Derby, USA

**Keywords:** drug reaction, eosinophilia, failure, hypersensitivity, trough level, vancomycin

## Abstract

Drug reaction with eosinophilia and systemic symptoms (DRESS) is a rare but potentially life-threatening multi-system disorder with a mortality rate of up to 10%, due to severe hypersensitivity drug reaction involving the skin and multiple internal organ systems. We emphasize the increasing prevalence of DRESS syndrome secondary to vancomycin use. A 79-year-old woman presented to the hospital with complaints of right upper quadrant pain, intense pruritis, and jaundice of one-week duration. She was on vancomycin and cefepime for six weeks after a wound infection, and both the medicines were withheld a week ago due to the increasing creatinine. She was afebrile with a pulse-94/min, blood pressure-92/46 mm of Hg, and respiratory rate-14/min. She had scleral icterus, diffuse maculopapular rash, generalized edema, right upper quadrant tenderness, and a positive Murphy’s sign. Investigations revealed hemoglobin-10.5 gm/dl, white blood cell count-16.0 K/uL, peripheral eosinophil count-1730 K/uL, alkaline phosphatase-2742 U/L, aspartate transaminase-612 U/L, alanine transaminase-674 U/L, total bilirubin-14.2 mg/dl with a direct component of 9.5mg/dl, blood urea nitrogen-64 mg/dl, creatinine-5.01 mg/dl, glomerular filtration rate-8 ml/min and vancomycin trough level-10.8 mcg/ml. Imaging studies were unremarkable. The renal function improved after high dose steroids, N-acetylcysteine and withdrawal of vancomycin, but the progression of liver failure continued. Eventually, she passed away due to multiorgan failure. Vancomycin is a rare drug to cause DRESS syndrome with 31 cases reported to date. Early recognition of this condition can hasten proper treatment and recovery. Further research on the association of vancomycin trough levels and DRESS syndrome needs to be conducted.

## Introduction

Drug reaction with eosinophilia and systemic symptoms (DRESS) syndrome is a severe hypersensitivity drug reaction involving the skin and multiple organs.

Vancomycin is rapidly excreted in the urine without significant hepatic metabolism. Hypersensitivity accounts for the instances of mild anicteric hepatitis associated with DRESS syndrome due to intravenous vancomycin. The association of alanine transaminase elevations with oral vancomycin is rare due to lack of oral absorption. Chen et al. in a meta-analysis showed an increased incidence of hepatic events, specifically elevated serum transaminase levels, in patients receiving vancomycin (6.8%) compared to those who were not (3.9%) [1].

The pathogenesis of DRESS syndrome is still unclear. In support of an immune mechanism, several drugs that cause DRESS syndrome have been linked with the human leukocyte antigen (HLA) haplotypes: allopurinol with HLAB*5801 [2]; carbamazepine with HLA-A*3101 in Japanese [3] and European patients [4]; and abacavir with HLA-B*5701 [5]. There may be a link between HLA alleles and DRESS syndrome secondary to vancomycin. There may also be an association with the underlying viral infection, such as human herpesvirus (HHV) 6 [6].

Vancomycin use is associated with nephrotoxicity with risk factors including the potential synergistic nephrotoxicity of vancomycin and piperacillin‐tazobactam. Oxidative stress is known to be the potential mechanism of nephrotoxicity. Agents inhibiting oxidative stress and reducing renal accumulation are thought to be protective. These include α‐lipoic acid, Ginkgo biloba extract, melatonin, vitamin C, vitamin E, N‐acetylcysteine, and curcumin (the main component of turmeric).

Symptoms typically present with a skin rash, which is usually diffuse and maculopapular, although other presentations, such as vesicles, bullae, pustules, cheilitis, purpura, target lesions and, erythroderma have been described. It is accompanied by fever, eosinophilia, atypical lymphocytosis, and multiple organ failure including the liver (may range from asymptomatic and mild transaminitis to fulminant liver failure), kidneys and lungs. Other manifestations include myocarditis/pericarditis, nephritis, acute respiratory distress syndrome, colitis, and encephalitis. Symptoms of DRESS syndrome appear after two to six weeks from the initial drug exposure suggesting a prolonged latency period. Mortality rates of up to 10% have been reported [7].

The most critical step in managing patients with DRESS syndrome is to identify the triggering drug and stop it. This can be difficult as hospital patients are often on multiple agents, the onset of symptoms is often delayed, and there is no validated diagnostic test. Vancomycin is rarely associated with DRESS syndrome, with 31 cases reported to date. There is an increase in reported cases in recent years, perhaps due to the increased use after the emergence of methicillin-resistant *Staphylococcus aureus* (MRSA), or a trend towards using continuous intravenous infusions leading to higher trough levels and higher total dosages of vancomycin. Although more intensive vancomycin administering schedules (including continuous infusions) are being used to achieve vancomycin trough levels of 15-20 mg/L and vancomycin trough levels >15 mg/L are an independent predictor of nephrotoxicity [8], any relationship with other vancomycin-associated adverse effects, including DRESS, have not been examined systematically in recent studies. We present this case to highlight vancomycin-induced DRESS as a severe and potentially life-threatening syndrome in the hospital setting.

## Case presentation

A 79-year-old Caucasian woman with a history of lumbar stenosis (status post lumbar laminectomy two months back, complicated by surgical site infection), gastroesophageal reflux disease, hyperlipidemia, hypothyroidism, presented to the hospital six weeks post-surgery with complaints of right upper quadrant pain, intense pruritis and jaundice of one-week duration. She was on prolonged antibiotic therapy with vancomycin and cefepime for six weeks for lumbar wound infection, both the medicines were stopped a week prior to the hospital visit due to worsening renal function. Her vitals were temperate of 37ºC, pulse of 94/minute, blood pressure of 92/46 mm of Hg, and respiratory rate of 14/minute. On examination, she had scleral icterus, diffuse maculopapular rash, right upper quadrant tenderness, a positive Murphy’s sign, and generalized edema.

**Figure 1:**
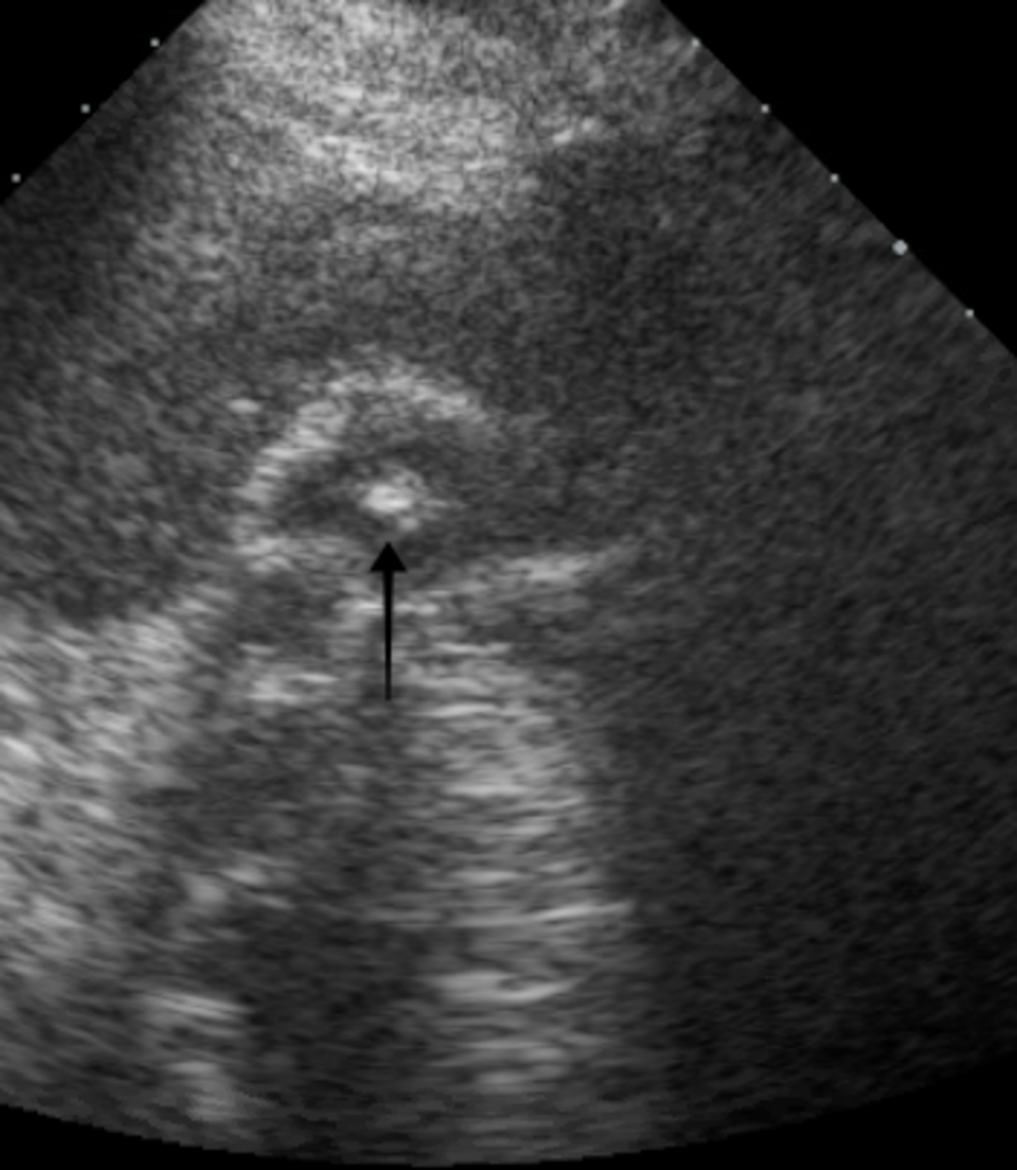
Ultrasonography of the abdomen revealing cholelithiasis

**Figure 2:**
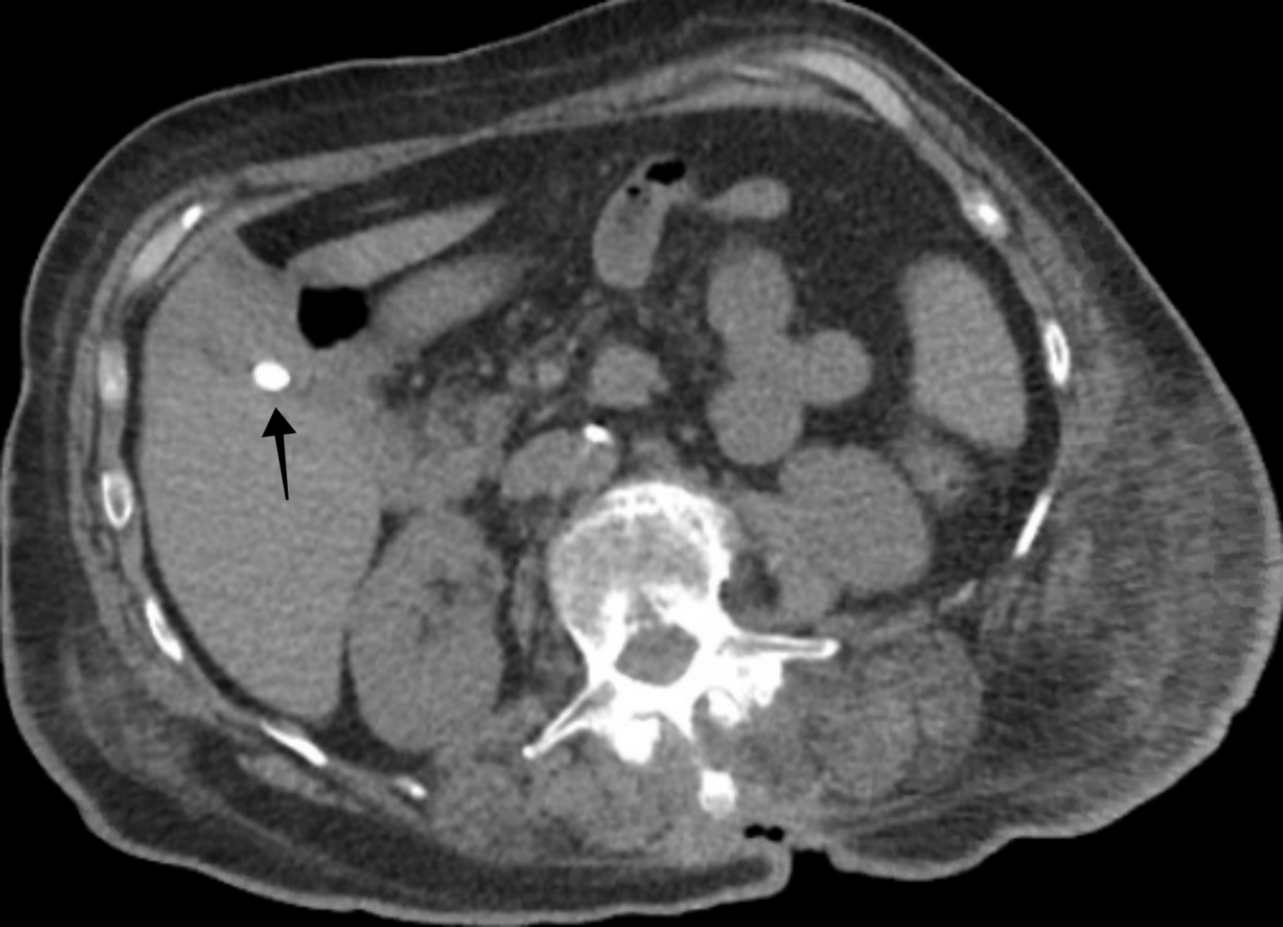
Computed tomography of the abdomen revealing cholelithiasis

**Figure 3:**
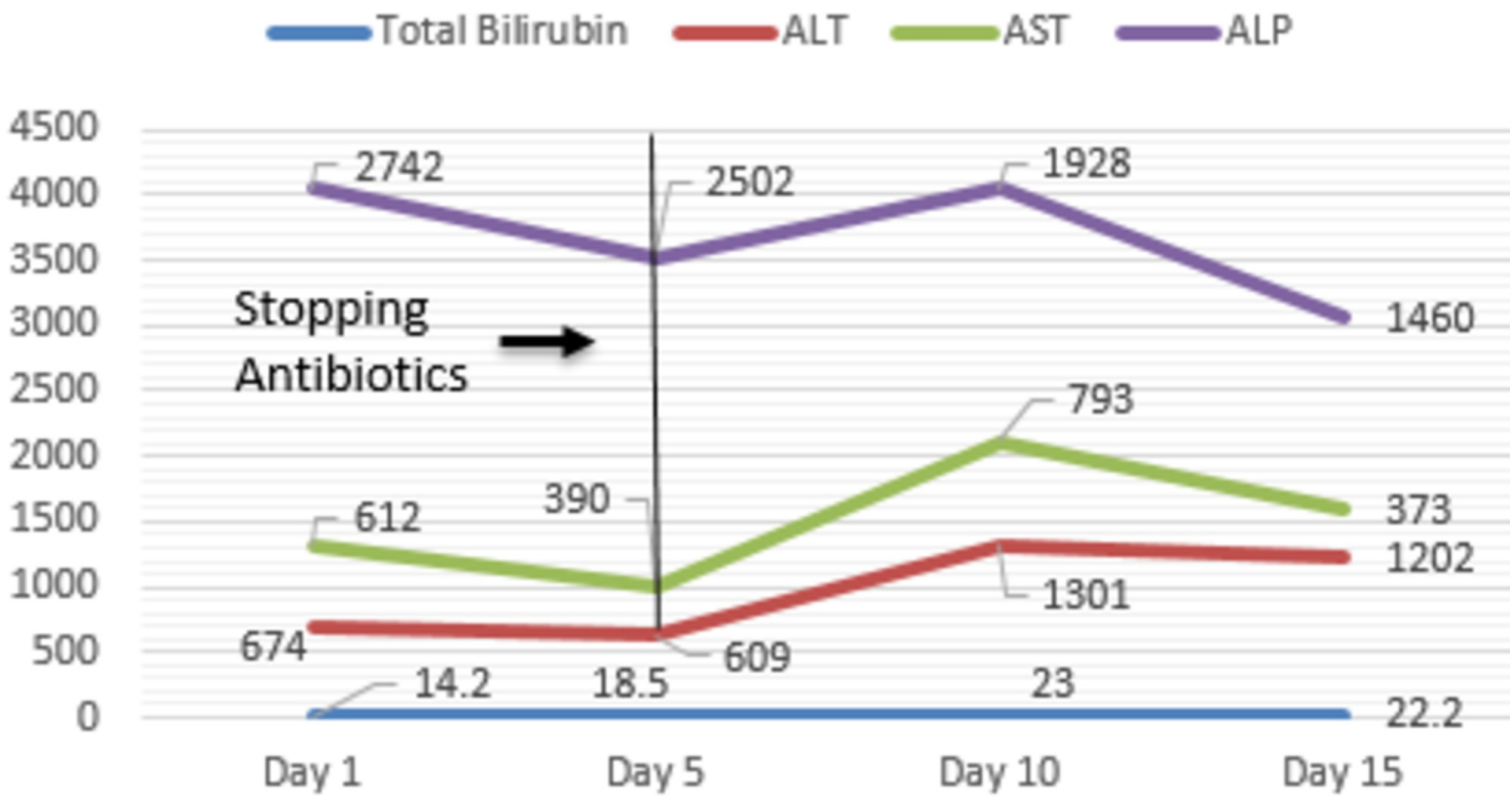
Trends of liver function tests during the hospital stay ALT-Alanine transaminase AST-Aspartate transaminase ALP-Alkaline phosphatase

**Figure 4:**
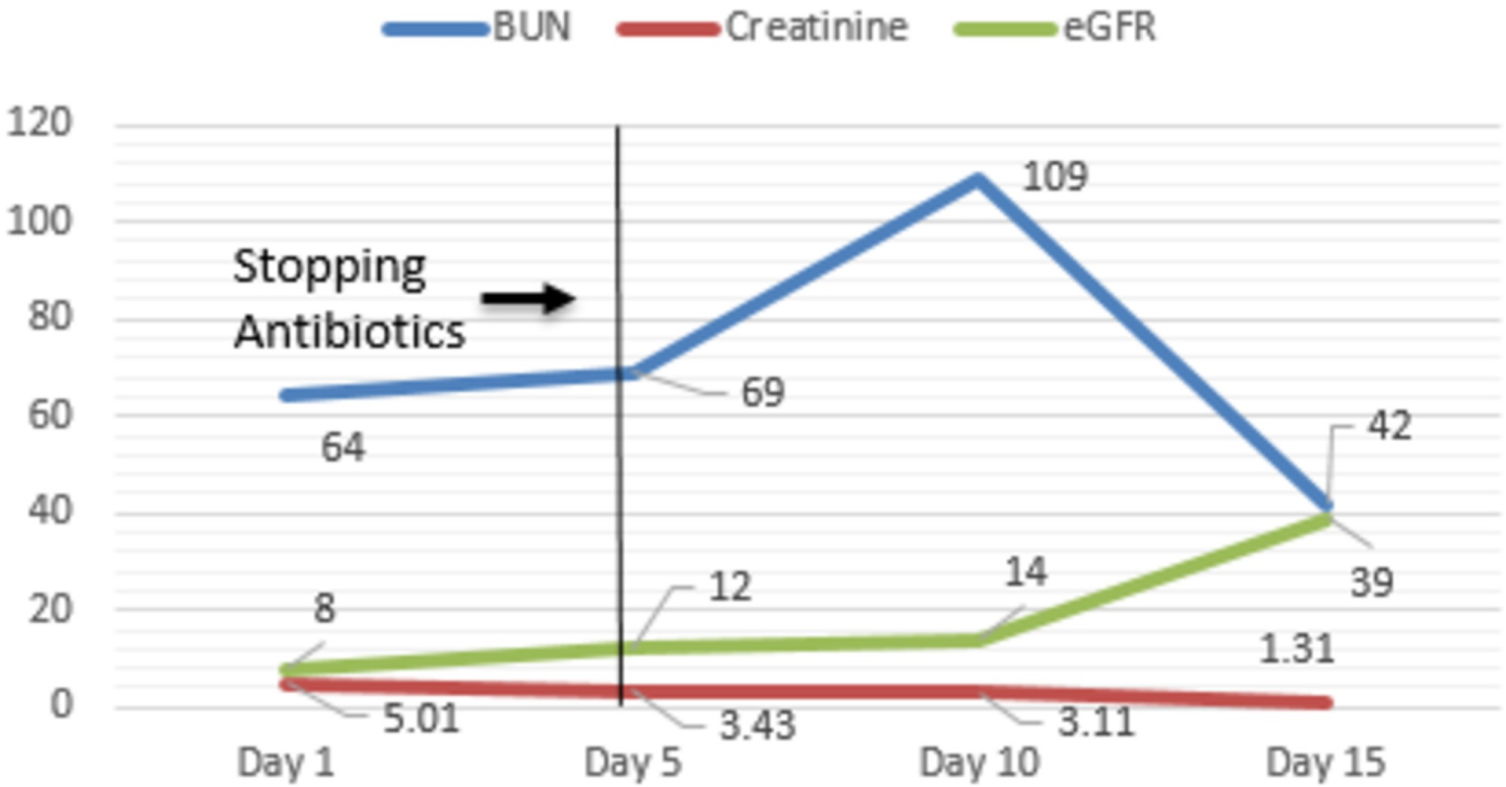
Trends of renal function tests during the hospital stay BUN- Blood urea nitrogen eGFR-Estimated glomerular filtration rate

Her investigations revealed a hemoglobin of 10.5 gm/dL, white blood cell count of 16.0 K/uL, peripheral eosinophil count of 1730 K/uL, alkaline phosphatase (ALP) of 2742 U/L, aspartate transaminase (AST) of 612 U/L, alanine transaminase (ALT) of 674 U/L, total bilirubin of 14.2 mg/dl with a direct component of 9.5mg/dl, blood urea nitrogen (BUN) of 64 mg/dl, creatinine of 5.01 mg/dl (with a baseline creatinine of 0.61 mg/dl), estimated glomerular filtration rate (eGFR) of 8 ml/min, and a vancomycin trough level of 10.8 mcg/ml. An ultrasound of the right upper quadrant of the abdomen revealed cholelithiasis with positive sonographic Murphy’s sign (Figure 1), computed tomography of the abdomen without contrast showed cholelithiasis with no inflammation and a common biliary duct of 4 mm in diameter (Figure 2). Magnetic resonance cholangiopancreatography was negative for obstruction. She was treated supportively with fluids and continued on vancomycin, as she met systemic inflammatory response syndrome criteria. Her vitals stabilized on day seven of hospital stay. Investigations revealed a hemoglobin of 8.4 gm/dL, white blood cell count of 30.4 K/uL, ALP of 2003 U/L, AST of 686 U/L, ALT of 971 U/L, total bilirubin of 22.2 mg/dl, BUN of 87 mg/dl, creatinine of 3.5 mg/dl, and eGFR of 11 ml/min. The trends of liver function tests and renal function tests are shown in Figures 3 and Figure 4, respectively. 

 

Given her recent antibiotic use, maculopapular rash, and eosinophilia in the setting of multi-organ failure, the diagnosis of DRESS syndrome was made. As per the European Registry of Severe Cutaneous Adverse Reaction Criteria (RegiSCAR) [9], the probability of vancomycin-induced DRESS syndrome was scored as “Definite.” Vancomycin was stopped, and she was started on high dose steroids (IV methylprednisolone 40 mg 8-hourly (0.5-2 mg/kg)) and N-acetylcysteine. The patient initially responded to steroids as indicated by an improvement in renal function, eventually developing progressive hepatic failure. Given the acuity of her condition, a renal biopsy was not indicated. She was not a good candidate for liver transplant given her age and comorbidities. She was sent to a long-term acute care facility with a plan of tapering steroids for 12 weeks. She eventually passed away due to multi-organ failure.

## Discussion

The syndrome of DRESS is a distinct, severe, idiosyncratic reaction to a drug characterized by a prolonged latency period. The clinical manifestations of DRESS syndrome include but are not limited to, fever, rash, lymphadenopathy, eosinophilia, and a wide range of mild-to-severe systemic presentations (Table 1) [10]. The diagnosis of DRESS syndrome is mostly clinical, but confirmation is through biopsy. The pathogenesis is still unclear. Treatment primarily involves the removal of the offending drug and supportive care. Systemic corticosteroids are used routinely, but their effectiveness is still unclear. 

**Table 1:** Drugs and Their Constellation of Manifestations Observed as DRESS Syndrome

Name of Drug	The Constellation of Manifestations Observed
Lamotrigine	Fever and toxic epidermal necrosis
Allopurinol	Dysfunction and eosinophilia without fever appearing several months after the start of treatment
Minocycline	Peripheral adenopathy, eosinophilia, heart abnormalities, and eosinophilic pneumopathy
Abacavir	Gastrointestinal and acute viral pneumonia-like symptoms of rapid occurrence after the introduction of treatment

Multiple diagnostic criteria have been developed and used to standardize the diagnosis and management of DRESS with limited success. The European Registry of Severe Cutaneous Adverse Reactions (RegiSCAR) suggested criteria for hospitalized patients with a drug rash to diagnose DRESS syndrome (Table 2) [11]. A Japanese group recommended another set of diagnostic criteria, which includes HHV-6 activation (Table 3) [12]. 

**Table 2:** RegiSCAR Criteria for The Diagnosis of DRESS

Criteria for Diagnosis
Hospitalization
Reaction suspected to be drug-related
Acute rash
Fever >38° C*
Enlarged lymph nodes at a minimum of 2 sites*
Involvement of at least one internal organ*
Blood count abnormalities*
Lymphocytes above or below normal limits
Eosinophils above the laboratory limits
Platelets below the laboratory limits
Three out of four asterisked (*) criteria are required for making the diagnosis.

**Table 3:** Japanese Group’s Criteria For The Diagnosis of DRESS

Criteria for Diagnosis
Maculopapular rash developing > three weeks after starting with the suspected drug
Prolonged clinical symptoms two weeks after discontinuation of the suspected drug
Fever > 38 °C
Liver abnormalities (alanine transaminase> 100 U/L)
Leucocyte abnormalities
Leukocytosis (>11 X 10^9^/L)
Atypical lymphocytosis (> 5%)
Eosinophilia (>1.5 x 10^9^ /L)
Lymphadenopathy
Human Herpesvirus 6 reactivation
The presence of seven criteria confirms the diagnosis

Using these diagnostic criteria, DRESS syndrome must be recognized at an early stage leading to the immediate withdrawal of the causative drug. It has been reported that earlier drug withdrawal leads to better prognosis [13]. There is little evidence to support the use of corticosteroids in DRESS syndrome [14]. Other modalities of treatment such as cyclosporine, N-acetylcysteine and intravenous immunoglobulins may also be used, but the evidence is lacking [7,15-17]. Family members of the patients should be informed of the diagnosis DRESS as it is inheritable [18-19]. 

## Conclusions

An iatrogenic cause for presenting symptoms such as ours should always be considered. Unexplained eosinophilia can often provide an essential clue to etiology. Physicians should be wary that a fever is not always indicative of infection, it could be drug-induced and thus, appropriate drug history is preeminent.
